# Does Forest Continuity Enhance the Resilience of Trees to Environmental Change?

**DOI:** 10.1371/journal.pone.0113507

**Published:** 2014-12-10

**Authors:** Goddert von Oheimb, Werner Härdtle, Dieter Eckstein, Hans-Hermann Engelke, Timo Hehnke, Bettina Wagner, Andreas Fichtner

**Affiliations:** 1 Institute of General Ecology and Environmental Protection, Technische Universität Dresden, Pienner Str. 7, 01737, Tharandt, Germany; 2 Institute of Ecology, Leuphana University of Lüneburg, Scharnhorststr. 1, 21335, Lüneburg, Germany; 3 Centre of Wood Sciences, University of Hamburg, Leuschnerstr. 91, 21031, Hamburg, Germany; 4 Forestry Department Sellhorn, Niedersachsen State Forestry Department, Sellhorn 1, 29646, Bispingen, Germany; 5 Albrecht-von-Haller Institute for Plant Sciences, University of Göttingen, Untere Karspüle 2, 37073, Göttingen, Germany; Chinese Academy of Sciences, China

## Abstract

There is ample evidence that continuously existing forests and afforestations on previously agricultural land differ with regard to ecosystem functions and services such as carbon sequestration, nutrient cycling and biodiversity. However, no studies have so far been conducted on possible long-term (>100 years) impacts on tree growth caused by differences in the ecological continuity of forest stands. In the present study we analysed the variation in tree-ring width of sessile oak (*Quercus petraea* (Matt.) Liebl.) trees (mean age 115–136 years) due to different land-use histories (continuously existing forests, afforestations both on arable land and on heathland). We also analysed the relation of growth patterns to soil nutrient stores and to climatic parameters (temperature, precipitation). Tree rings formed between 1896 and 2005 were widest in trees afforested on arable land. This can be attributed to higher nitrogen and phosphorous availability and indicates that former fertilisation may continue to affect the nutritional status of forest soils for more than one century after those activities have ceased. Moreover, these trees responded more strongly to environmental changes – as shown by a higher mean sensitivity of the tree-ring widths – than trees of continuously existing forests. However, the impact of climatic parameters on the variability in tree-ring width was generally small, but trees on former arable land showed the highest susceptibility to annually changing climatic conditions. We assume that incompletely developed humus horizons as well as differences in the edaphon are responsible for the more sensitive response of oak trees of recent forests (former arable land and former heathland) to variation in environmental conditions. We conclude that forests characterised by a long ecological continuity may be better adapted to global change than recent forest ecosystems.

## Introduction

Forest ecosystems provide important ecological, social and economic services. They host much of the world’s biodiversity and are key systems in stabilising biogeochemical cycles and climate conditions at regional and global scales [1]. However, changes in land use and deforestation have caused a worldwide and extensive decline of forest ecosystems [2]. In western Europe and North America, in particular, a range of actions were taken in the 19^th^ and 20^th^ centuries to prevent deforestation and to establish new forests, mainly on former agricultural or degraded land [3], [4]. In recent years, the moves to reduce atmospheric CO_2_ levels have provided a strong impetus to expand the area covered by forests worldwide [5].

Despite the multiple positive effects of an increase in forest area, many questions regarding the evaluation of the overall ecological implications of such an afforestation policy remain open [6], [7]. There is ample evidence that the legacies of former land-use activities may continue to affect forest ecosystem functioning for decades or even centuries after those activities have ceased [8], [9]. Significant differences in plant species composition and diversity as well as in soil properties between areas continuously covered with forests for several centuries, i.e., sites with a long ecological continuity ([10]; also termed “ancient forests” [9]) and forests restored on former agricultural land have been extensively documented [11], [12], [13], [14], [15], [16], [17], [18], [19], [20]. In addition, numerous studies have reported impacts of land-use changes on soil microbial communities, microbial community function and, consequently, nutrient cycling rates [21], [22], [23], [24], [25]. Thus, over a long period of time, forests restored on former agricultural land may differ from continuously existing forests with regard to their ecosystem properties and desirable ecosystem services. Surprisingly, little information is available on the long-term consequences of land-use change on individual tree growth. To our knowledge, the present study is the first to use a dendroecological approach to compare long-term radial growth of sessile oak (*Quercus petraea* (Matt.) Liebl.) trees in continuously existing forests and afforestations on previously agricultural land.

Radial growth of trees is related to abiotic and biotic factors, tree age and forest management. Among the local abiotic site factors, soil fertility and soil water availability, most important for oak diameter growth [26], [27], [28], can be affected by past land-use. Past fertilisation can increase the soil pools of N and P in forests on former agricultural land [15], [29], [30], [31]. For example, Baeten et al. [32] showed that the growth performance of forest herbaceous species can be enhanced by increased P availability in post-agricultural forests. Cultivation procedures (tillage and fertilisation) reduce the thickness of the humus horizons or even cause them to disappear. After abandonment and reforestation, the forest floor redevelops, though at a slow rate. Von Oheimb et al. [15] predicted that a minimum of 250 years must pass before the forest floor C stores, typical for continuously existing oak forests, have accumulated on former arable land. Thinner and less developed humus horizons are accompanied by a deterioration of the water storage capacity of the soils [33]. High temperature and water stress caused by low precipitation during spring and summer as well as early and late frost events are the main influences on the climate-growth relationships of oak. However, under sub-oceanic and sub-continental conditions the sensitivity of sessile oak radial growth to climate was found to be generally low [34], [35], [36], [37], [38]. Furthermore, interactions between below-ground and above-ground communities may strongly affect tree growth, because soil microbial communities regulate key biogeochemical processes such as decomposition of organic materials, carbon and nitrogen cycling, and nutrient availability for plant growth [39], [40], [41]. There is a growing body of evidence that different land-use practices alter soil communities in the long term [21], [23], [25].

In this study we analysed the variability in growth of mature oak trees growing in continuously existing forests and in afforestations on former arable land and on former heathland. We expected tree-ring width of oak to be highest on former arable land, mirroring the after-effects of past fertilisation. We hypothesised that oak trees growing on continuously existing forest sites show a lower variability in their tree-ring width compared to those on afforested sites, indicating that they are less responsive to varying environmental conditions.

## Materials and Methods

### Study area

The study area is the nature reserve Lüneburg Heath (NW Germany, 53°15′N, 9°58′E, 60 m a.s.l.), comprising an area of 234 km^2^ with soil and climate conditions representative of most parts of NW Central Europe. The climate is of a humid sub-oceanic type. Mean annual sum of precipitation is 811 mm and mean annual temperature is 8.4°C [42]. Soils consist of deposits of the Saale Ice Age. The predominant soil types are Podzols.

In the study area both the land-use and forest history have been well-documented over the last 230 years [43]. In 1776, almost 80% of the area was covered by heathlands dominated by *Calluna vulgaris*, whilst agricultural fields and forests occupied about 10 and 5%, respectively. In the middle of the 19^th^ century, farmers began to abandon heathlands and arable land and vast areas were afforested or underwent natural succession. Today, forests span about 60% and heathlands about 20% of the landscape.

### Site selection

In the nature reserve, a total of 25 discrete sample sites dominated by *Quercus petraea* (1–4 ha in size and embedded in surrounding stands of *Pinus sylvestris*, *Picea abies*, or *Fagus sylvatica*) were chosen using recent forest maps and historical maps (including the so-called “Kurhannoversche Landesaufnahme” from 1776–1786). The historical maps indicate the type of land-use during the past 230 years and thus served to detect continuously existing forests (hereafter referred to as “CEF”). It is assumed that these woodlands have never been deforested in the last 200 to 300 years, but used as woodland pastures [43]. Forest management plans from 1878 were used to detect oak stands in areas which had been arable land or heathland (hereafter referred to as “FAL” and “FH”, respectively) prior to this date (Table 1). In addition, plans gave information about tree age and the silvicultural measures which were implemented from 1878 onwards. The age of the study trees was, on average, 124 years on FAL, 115 years on FH, and 136 years in the CEF. In recent decades, selective logging but no soil disturbance occurred on the sample sites.

**Table 1:** Site land-use history and characteristics.

	Former arable land	Former heathland	Continously existing forest
Historical management activities	Ploughing,	Sheep grazing,	Firewood colection,
	application of manure	sod-cutting	litter ranking
No. sample sites	10	8	7
Stand age (years)	124	115	136
Stand volume (m^3^ ha^−1^)	295	257	346
Species composition (%)^A^			
* Quercus petraea*	86	80	67
* Fagus sylvatica*	2	-	25
* Pinus sylvestris*	6	15	4
* Picea abies*	6	3	4
Other tree species	-	1	-
Site index^B^	3–4 (mesothrophic)	3–4 (mesothrophic)	3–4 (mesothrophic)
HG (r)	0.69^ns^	0.67^ns^	0.78^ns^
TRW (mm)	1.8^a^	1.7^ab^	1.5^b^
SD (mm)	0.61^b^	0.41^a^	0.37^c^
AR-1	0.77^ns^	0.56^ns^	0.64^ns^
MS	0.17^a^	0.17^a^	0.14^b^

A Mean proportion of canopy tree basal area according to the forest management plan of 2011.

B The soil nutrient status of the sample sites was classified according to the German forest site mapping system. This index ranges from 1 (very low nutrient availability) to 6 (very high nutrient availability).

Structure and growth parameters (means) of sessile oak (*Quercus petraea*) stands in the nature reserve Lüneburg Heath (NW Germany) for the period from 1896 to 2005. Number of trees per sample site: 10. Abbreviations: HG = homogeneity of growth; TRW = tree-ring width; SD = standard deviation; AR-1 = autocorrelation; MS = mean sensitivity. Different superscript letters indicate significant differences of tree ring series characteristics among the historical land-use types (*P*_adj._<0.05); ns = not significant.

### Tree selection and sampling design

The research permission was provided by the Forestry Department Sellhorn, Niedersachsen State Forestry Department, Bispingen, Germany. No specific permissions were required for our activities. Our field studies did not involve any endangered species.

The dendroecological sampling was carried out from June to August 2006. For the selection of the study trees a grid was laid over each sample site. In this grid, 10 intersections were chosen randomly and the dominant oak tree nearest to an intersection was selected ( = 10 trees per sample site). A total of 250 trees were sampled. Radial growth was derived from two cores per tree taken at breast height (1.30 m) using an increment borer (Suunto 400, Vantaa, Finland; 40 cm in length and 0.5 cm in bit diameter).

### Tree-ring analysis

**Figure 1:**
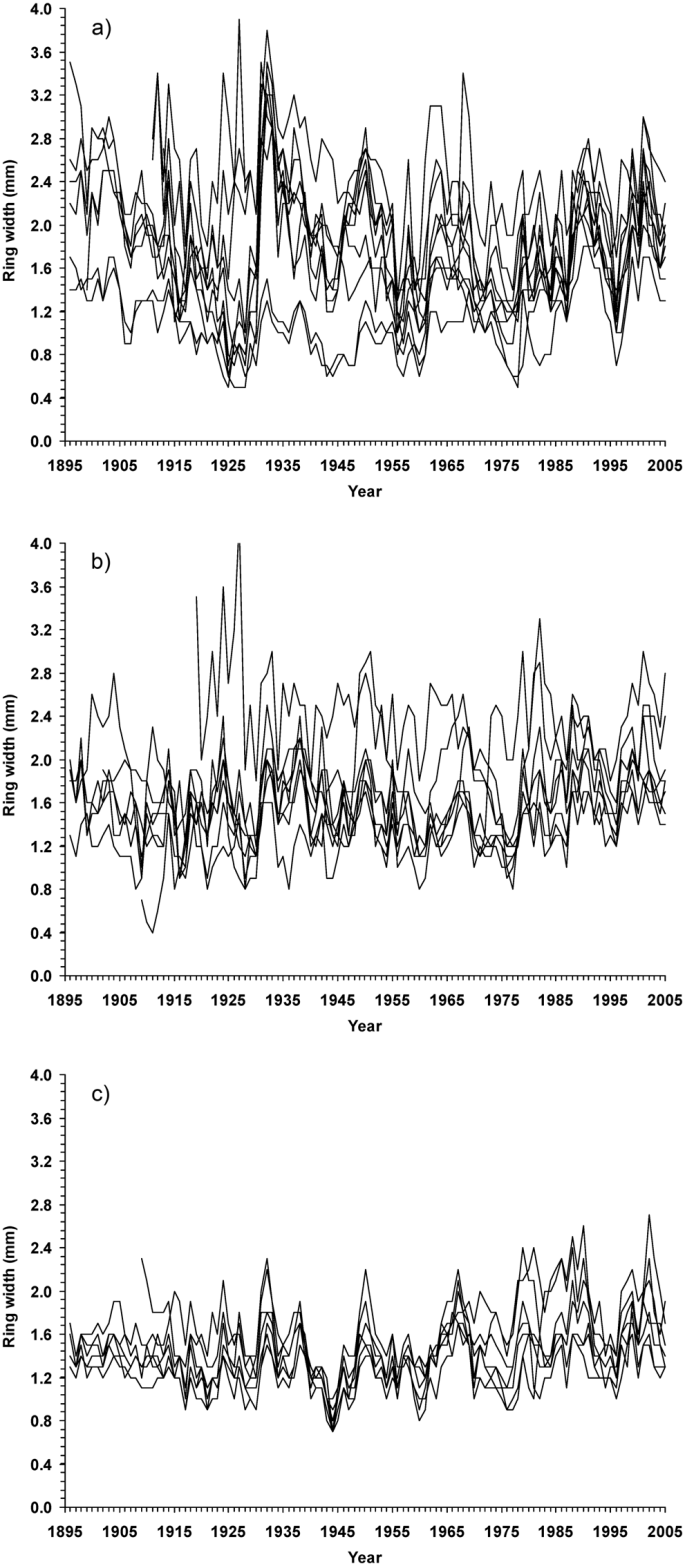
Variation of radial growth of sessile oak (*Quercus petraea*) among historical land-use types between 1896 and 2005. Data represent site chronologies based on 10 trees per sample site; a) former arable land (10 sites), (b) former heathland (8 sites), and (c) continuously existing forests (7 sites).

**Figure 2:**
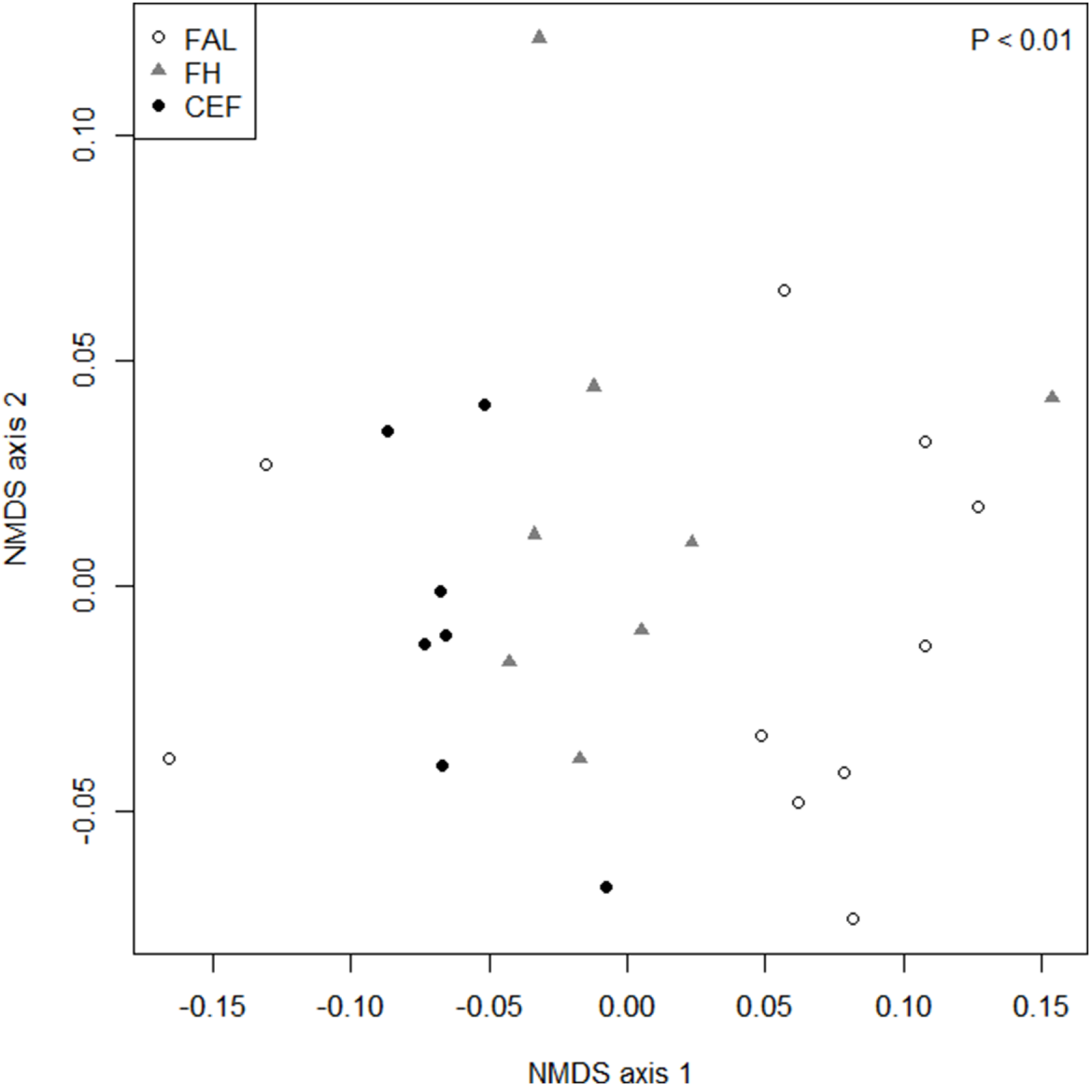
Similarity in temporal variation of growth rates in response to past land-use. Non-metric multi-dimensional scaling ordination (stress: 0.10) of site chronologies (period 1896–2005) of sessile oak (*Quercus petraea*) growing in oak forests with different land-use histories: Former arable land (FAL), former heathland (FH) and continuously existing forests (CEF). Site scores represent mean tree-ring width series derived from 10 trees per site.

The cores were air dried, fixed to a core-mounting, and their surface was smoothed with a core-microtome (WSL Birmensdorf, Switzerland). Subsequently, tree-ring widths (TRW) were measured using a measuring table with 0.01 mm resolution (Instrumenta Mechanik Labor IML, Wiesloch, Germany) combined with a binocular (Wild, Heerbrugg, Switzerland) and recorded using the IML software T-Tools pro. The data were analysed using the software TSAP-Win (Version 0.53, Rinntech, Heidelberg, Germany). First, the two TRW series per tree were cross-dated and averaged into a single tree-ring series. Then, an average series per sample site (site chronology) and finally per historical land-use type (land-use type chronology) were calculated. Due to differences in tree age within and between sites, the further analyses were confined to the common period from 1896–2005. The raw TRW data were used to visualize land-use type-specific differences in radial increment (cf. Figs. 1 and 2; raw TRW data in Table S1). For the analysis of TRW-climate relationships, the raw TRW data have been detrended (see description below).

### Soil and meteorological data

Soil data were taken from an accompanying study that analysed soil parameters appropriate for an assessment of the nutritional status of the sample sites (Table 2) [15]; there are significant differences between the soil parameters for the three historical land-use types. Furthermore, patterns differed for the respective soil horizons (i.e. organic layer and A-horizon). FAL showed significantly lower C and N contents in the organic layer. The C/N ratios (in the O- and A-horizon) were lowest on FAL and highest in the CEF. In addition, both plant-available P and total P contents were highest in the A-horizon of FAL, but were of a similar order of magnitude in FH and CEF. High P contents were reflected by low C/P ratios of this horizon on FAL.

**Table 2:** Soil ecological properties of the three historical land-use types (means and SD; n = 10 sample sites per site type; all data from von Oheimb et al. [15]).

Soil parameters	Soil horizon	Former arable land		Former heathland		Continuously existing forests	
C-content (%)	O	9.27^b^	(1.26)	19.17^a^	(2.00)	18.80^a^	(2.43)
	A	2.01	(0.20)	2.19	(0.68)	2.39	(0.57)
N-content (%)	O	0.47^b^	(0.06)	0.88^a^	(0.09)	0.80^a^	(0.10)
	A	0.11	(0.01)	0.10	(0.03)	0.08	(0.02)
C/N-ratio	O	18.8^b^	(0.3)	21.5^a^	(0.8)	23.6^c^	(0.6)
	A	20.2^b^	(1.1)	26.8^a^	(1.9)	31.2^a^	(1.9)
P_pa_ (mg L^−1^)	O	6.9	(1.2)	6.5	(0.5)	8.9	(1.7)
	A	23.9^b^	(3.8)	6.0^a^	(1.6)	9.0^a^	(3.3)
P_t_ (mg L^−1^)	O	61.5^b^	(5.3)	102.8^a^	(12.8)	104.5^ab^	(17.2)
	A	261.9^b^	(27.0)	143.5^a^	(46.5)	140.7^a^	(44.5)
C/P-ratio	O	218.9^b^	(23.9)	286.3^a^	(18.4)	287.1^a^	(19.1)
	A	107.5^b^	(14.9)	217.0^a^	(18.7)	237.9^a^	(19.8)
Base saturation (%)	O	35.2^a^	(5.3)	35.5^a^	(4.3)	24.7^b^	(1.7)
	A	34.8	(7.1)	36.7	(6.1)	23.1	(5.3)
CEC (mval L^−1^)	O	21.4^b^	(2.4)	30.3^a^	(2.8)	39.6^c^	(3.9)
	A	7.3	(0.9)	7.2	(1.7)	8.6	(2.0)
pH_H2O_	O	4.2	(0.1)	4.1	(0.2)	4.0	(0.1)
	A	4.0	(0.1)	4.1	(0.1)	4.0	(0.1)

Soil parameter abbreviations: P_pa_ = plant available phosphorous; P_t_ = total P-content; CEC = cation exchange capacity; O = organic layer, A = A-horizon (i.e. upper mineral horizon). Different superscript letters indicate significant differences of a given parameter among the three historical land-use types (*P*_adj._<0.05).

Meteorological data (mean monthly precipitation and mean monthly temperature, available from 1862 onwards) were obtained from the German Weather Service (DWD, Hamburg, Germany) and from the Hamburger Bildungsserver (HBS, Hamburg, Germany) and are from the weather station at Wilsede (Lüneburg Heath), located within the study area. The meteorological data for the study period 1896–2005 are given in Tables S2 and S3.

### Data analyses

The homogeneity of growth within each land-use type is expressed as the mean Pearson’s correlation (r) among the respective TRW site chronologies. Descriptive statistics for TRW series (mean tree-ring width, standard deviation, first-order autocorrelation, and mean sensitivity) were calculated using TSAP-Win. The mean tree-ring width ± standard deviation allows a comparison of radial growth rates between the land-use types. The first order autocorrelation (AR-1) indicates the influence of the previous year’s growth on the tree-ring width of the current year, and the mean sensitivity (ms) is a measure of the year-to-year variability in tree-ring width ranging from 0 to 1 [44], [45]. Differences in these statistical parameters (log-transformed) between the historical land-use types were tested for significance by a one-way analysis of variance (ANOVA) followed by a post-hoc performance (Tukey’s HSD test).

The temporal variation in growth (period 1896–2005) depending on the land-use type was evaluated by an analysis of similarity (ANOSIM; [46]), performed on a matrix of Bray-Curtis dissimilarities based on the raw TRW site chronologies. To display differences in growth between and within land-use types, we used non-metric multi-dimensional scaling (NMDS, using the *metaMDS* function of the *vegan* library in R; [47]).

The relationship between TRW and soil chemistry was assessed by Pearson’s correlation, using TRW site chronologies (from 1896–2005) and soil data. Due to a lack of soil data for four sample sites, the analysis was performed with 21 sample sites (data in Table S4). Climate impacts on TRW were analyzed by means of multiple linear regressions (following the approaches as described in Härdtle et al. [38], [48]). To this end, we detrended the raw TRW data of single trees using the residuals from five-year moving averages (TSAP-Win). This procedure removes long-term trends such as age effects but keeps the high-frequency (i.e. inter-annual) signals typical of a respective chronology [38], [48], [49], [50]. Then, the single tree-specific chronologies were averaged to site-specific chronologies. The subsequent regression analyses were based on these site chronologies. In the regression analyses, detrended TRW was considered as the dependent variable. Monthly precipitation and temperature from July of the previous year to August of the current year were used as the physiologically most meaningful predictors [37], [51]. In addition, we included precipitation totals of the previous and current growing season (April-October), mean annual temperature (previous and current year), and previous year’s TRW as further predictors of radial increment. The climatic variables were detrended in the same way as the ring-width data. Model selection was based on the identification of significant (*P*<0.05) predictor variables. A correction for the degrees of freedom was applied considering autocorrelation (AR-1) between current and previous year’s TRW (effective sampling size N’ = N (1−(AR-1)) (1+(AR-1))^−1^ [52]. Regression analyses were carried out using the SPSS 20.0 package (SPSS Inc., Chicago/IL, US).

## Results

There was a trend towards higher but more variable growth rates at afforested sites. The homogeneity of growth within each of the three historical land-use types was highest for CEF (r = 0.78) followed by FAL (0.69) and FH (0.67) (Table 1; Fig. 1).

The mean TRW was significantly higher on FAL (1.8 mm) than in CEF (1.5 mm) (Table 1); on the FH an intermediate TRW was found (1.7 mm). Likewise, the mean standard deviation of the series declined significantly from FAL to FH to CEF. The first order-autocorrelation was high for all three land-use types but not statistically different between them. The mean sensitivity was significantly higher for trees of FAL and FH (both 0.17) than for trees of the CEF (0.14).

Tree-ring widths mirrored differences in soil chemistry, as increased TRW coincided with the higher nutrient levels found for the FAL (Table 3). Low C/N ratios (O-horizon and A-horizon), higher amounts of plant-available P and total P contents (A-horizon), and a higher base saturation (O-horizon) were significantly correlated with increased tree-ring widths. No relationship was found between tree-ring widths and soil pH values.

**Table 3:** Pearson’s correlations between mean tree-ring widths (of sample sites; n = 21) and soil parameters.

Parameter	Soil horizon	r
C-content (%)	O	−0.46*
	A	ns
C/N-ratio		−1
	A	−0.56**
P_pa_ (mg L^−1^)	O	ns
	A	0.61**
P_t_ (mg L^−1^)	O	ns
	A	0.57**
C/P-ratio	O	ns
	A	−0.70**
Base saturation (%)	O	0.44*
	A	ns

Only significant correlations (* = *P*<0.05, ** = *P*<0.01) with r>|0.5| are considered; ns = not significant; period of tree-ring analyses: 1896–2005. For abbreviations of soil parameters see Table 2.

The first NMDS axis clearly separated the site chronologies (Fig. 2), indicating that the temporal variation in growth rates were driven by land-use legacies (*R = *0.240, *P*<0.01). TRW chronologies of CEF sites differed markedly from FAL (*R = *0. 383, *P*<0.01), while differences between CEF and FA were less distinct (*R = *0.159, *P*<0.05). A marginal difference was noted between FAL and FA (*R = *0.164, *P*<0.05). Moreover, within a land-use type the variability was lowest for CEF (highest similarity of site chronologies; Fig. 2), suggesting a trend towards a more balanced growth response with increasing forest continuity.

Trees on FAL showed the highest susceptibility to shifts in climatic conditions. However, the overall responses of TRW to climatic variability were low across land-use types (variance explained: FAL = 20%, FH = 7%, CEF = 6%; Table 4). With the exception of FH, the previous year’s TRW was a significant predictor for the current year’s radial increment. On FAL, oak trees responded negatively to high spring (March, May) temperatures, but positively to mild conditions in February. Summer (June, July) precipitation was negatively correlated with the TRW of oak on FH and CEF.

**Table 4:** Multiple linear regression analyses (stepwise forward selection) of effects of climatic variables on tree-ring width (TRW) of sessile oak trees (n of sample sites = 25).

Dependent variable	TRW		
Predictors	Former arable land	Former heathland	Continuously existing forests
Previous year’s TRW	0.24**		0.22*
Precipitation June		−0.21*	
Precipitation July			−0.19**
Temperature February	0.43**		
Temperature March	−0.65***		
Temperature May	−0.25**		
Temperature June	0.23**		
Temperature November_prev_		−0.18*	
R	0.48	0.29	0.27
R_adj_^2^	0.20	0.07	0.06
P	<0.001	0.005	0.011
df_Resid_	100	117	84

Values denote the partial correlation coefficient (beta) for each regression model. Significance of predictors: * = *P*<0.05, ** = *P*<0.01, *** = *P*<0.001; “prev.” as subscript character refers to climatic data of the previous year of tree-ring formation.

## Discussion

As hypothesized, the variability of TRW of oak trees tended to be higher in afforestations (FAL, FH) than in CEF. The low standard deviation and mean sensitivity of TRW in CEF suggest that these trees are less susceptible to shifts in environmental conditions than trees in afforestations. However, the underlying mechanisms that may explain this finding have not been studied so far. In principle, a number of different mechanisms are conceivable.

### Relationships between growth variability and soil conditions

In CEF, humus horizons such as organic layers are well-developed and significantly thicker than in afforestations [15]. This is accompanied by an improved water storage capacity, which in turn can mitigate the effects of a severe drought during the growing season [33]. Thus, well developed humus horizons may also dampen adverse effects of increasing or more severe drought events related to climate change. Organic layers are also responsible for minimising fluctuations in soil temperature and of the moisture of the A-horizon [53]. In addition, the thickness of the humus horizons and the soil profile impact root distribution patterns of oak trees [54]. Increasing thickness of humus horizons increases the soil volume that can be exploited by roots and thus improves the potential availability of water and nutrient resources. This applies to acid sites in particular, as here the bulk of fine and coarse roots are concentrated in the humus horizons [55].

Tree growth is also related to soil properties such as soil nutrient cycles that are known to be affected by historical land-use practices in the long term [8]. Improved nutrient conditions were the main reason for significantly increased tree-ring widths of oak trees growing on FAL. This result can be attributed, in particular, to enhanced N availability as expressed by significantly lower C/N ratios. C/N ratios are an important indicator of litter decomposition and of N mineralisation rates, and high C/N ratios are related to a low soil biological activity [56]. Accordingly, tree-ring widths were strongly negatively correlated with C/N ratios (−0.64, P<0.01; Table 2). Generally, tree-ring width of oak trees responds positively to increasing N availability. This applies to acid sites in particular [28], [57], since N proved to be the most growth-limiting nutrient in acid soils [54]. Positive responses of tree-ring width to N supply have been observed in both young and old oak stands [58], and may explain the fact that radial growth on FAL was accelerated over the entire time span investigated, 1896–2005. However, it is likely that the continuous increase in tree-ring widths across sites which was evident from the 1970s onwards (see Fig. 1) can be attributed to the increasing rates of atmospheric N loads found in NW Europe [38], [59]. Besides N, P supply and base supply may be co-limiting factors for the growth of oak on strongly acid soils [58]. This may explain significant correlations between tree-ring widths and soil parameters which describe the availability of P and base cations.

Furthermore, there are distinct differences in soil microbial communities between afforestations (FAL, FH) and CEF. In the studied forests, Fichtner et al. [25] found a persistent (>100 years) and significant variation in microbial community structure and microbial extra-cellular enzyme activity based on land-use history. Arbuscular mycorrhizal fungi, actinobacteria, and enzyme activities were distinctly lower in soils of CEF compared to former agricultural land. In contrast, microbial communities in soils of CEF were associated with the highest abundance of saprotrophic and ectomycorrhizal fungi and the highest structural heterogeneity. Furthermore, a high abundance of gram-negative bacterial markers was observed in southern Appalachian (USA) forests established more than 50 years ago on formerly cultivated land, whereas CEF were characterised by high levels of fungal and gram-positive bacterial markers [21]. Consequently, a long forest continuity may lead to more diverse microbial assemblages and more complex mycorrhizal systems. This may help to buffer growth fluctuations in high growth and low growth years. Historical land-use activities may, thus, have long-term effects on soil properties of forest ecosystems that need to be taken into account (as “historical site factor”) when analysing forest ecosystem processes [15], [25].

### Relationships between growth variability and climatic conditions

Oak trees in our study displayed a low sensitivity of radial increment towards changes in climatic conditions across the different historical land-use types. This is reflected by the low proportion of variance in TRW explained by climatic variables (20, 7, and 6% for FAL, FH, and CEF, respectively). Our finding is in agreement with other studies, according to which climatic variables proved to be weak predictors for tree-ring widths of oak in central Europe [34], [36], [37], [38], [60]. Instead radial growth is largely controlled by non-climatic factors such as soil conditions and biotic stressors (e.g. insect infestation; [26], [35], [61]). However, at the northern or southern range margins of oak in Sweden or Slovenia, respectively, the climatic signal becomes stronger [62], [63]. Even extreme climatic events (such as the summer drought in 2003) are weakly mirrored in radial increment rates of *Quercus petraea* [64], [65], and climatic extremes may appear to cause oak decline only in combination with other stress factors (e.g. defoliation resulting from insect infestation, infection with pathogenic fungi; [61]).

However, the proportion of variance in TRW explained by climatic variables was highest for oak trees on formerly ploughed soils (20% on FAL sites), suggesting a higher climatic susceptibility of FAL trees in comparison to trees grown at the other sites. At FAL sites, high temperatures in spring (March, May) negatively affected TRW in particular, which is in agreement with Mérian et al. [37]. Moreover, the current year’s increment rates do not only reflect the current year’s growing conditions, but they also depict an integrative response to the environmental conditions a tree experienced in the course of the previous year (at least for trees on FAL and in CEF). This coincides with findings of Becker et al. [66], who identified current year’s climatic variables as weak predictors for increment rates of *Quercus petraea*. Our results demonstrate that the overall growth response of sessile oak trees in relation to changes in climatic conditions is weak, but trees growing on CEF sites exhibit a particularly low susceptibility to climatic variability compared to trees that have been afforested on FAL.

### Relationships between growth variability and stand structure

The interpretation of our results also needs to consider environmental parameters that have not been quantified in our study. As oak species are known to be light-demanding trees, more favourable light conditions may have promoted tree-ring widths at afforested sites [67]. However, we consider the effects of the light conditions to be circumstantial in our study, because tree-ring widths of trees on FH did not differ significantly from those of CEF, despite potentially higher insolation rates on the heathland sites. We also exclude logging as a potential factor responsible for the differences in tree-ring width, since all sample sites experienced similar management intensities from a long-term perspective (according to the forest management plans of the nature reserve). Moreover, variation in species composition can alter local neighbourhood interactions. A high proportion of allospecific neighbours is assumed to benefit the growth rates of individual trees due to niche complementarity [68], [69]. However, the temporal variation of growth rates are more distinct in mixed-species neighborhoods compared to monospecific stands [70]. Consequently, a higher dissimilarity in the growth pattern should be obvious for CEF (lowest proportion of conspecifics; Table 1). In contrast, tree-ring chronologies in CEF were associated with the highest growth synchronization (Fig. 1). We therefore conclude that differences in the mode of tree neighborhood effects (intra- versus interspecific competition) play a minor role in explaining the growth pattern observed. The same applies to factors such as insect or fungi infestation that might have caused short-term decreases in growth rates. Severe damage of trees from insect or fungi infestation resulting in high tree mortality has not been reported for the study area. As afforestations were carried out using seed or saplings from the same provenances, growth differences between trees were also unlikely to result from different genotypes [71].

## Conclusions

Our study provides evidence that trees of recent forests tend to be more susceptible to shifting environmental conditions, as indicated by a higher mean sensitivity of tree-ring widths. “Historical site factors” are, thus, important for a deeper understanding of ecosystem functionality, since legacies resulting from historical land-use may still impact present-day patterns of tree growth. It is, therefore, likely that forests characterised by high ecological continuity are better adapted to global change than recent forest ecosystems.

These findings have important implications for the evaluation of current afforestation and nature conservation policies. Next to their outstanding role in preserving a high typical above- and belowground species diversity and functionality, sites with a long continuity (several hundred years) of forest cover may increasingly become more important in the context of ecosystem services (e.g. [31]). Consequently, at such sites of conservation priority, high-impact management measures like clear-cuts, tillage, fertilization or altering the natural vegetation by planting exotic tree species may negatively affect plant-soil interactions, and thereby reducing forest resilience with respect to environmental fluctuations. Implementing ecological continuity in forest management and conservation policies would potentially improve long-term ecosystem management approaches under changing environmental conditions.

## Supporting Information

Table S1 **Site chronologies of sessile oak (** ***Quercus petraea*** **) between 1896 and 2005 (tree-ring width in 1/10 mm).**
(PDF)

Table S2 **Mean monthly precipitation and precipitation total of the growing season (April–October, rainvp) (in mm) at the weather station Wilsede (Lüneburg Heath, NW Germany) for the period 1896 to 2005.**
(PDF)

Table S3 **Mean monthly temperature and mean annual temperature (mean temp) (in °C) at the weather station Wilsede (Lüneburg Heath, NW Germany) for the period 1896 to 2005.**
(PDF)

Table S4 **Mean tree-ring width (TRW) of sessile oak (** ***Quercus petraea*** **) during the period 1896–2005 (in 1/10 mm) and soil parameters.**
(PDF)
