# Supplementary material for: Does Forest Continuity Enhance the Resilience of Trees to Environmental Change?
Source: PLoS One. 2014 Dec 10;9(12):e113507. doi: 10.1371/journal.pone.0113507 (PMC4262476; doi:10.1371/journal.pone.0113507)
Supplement: Table S1 — Site chronologies of sessile oak ( Quercus petraea ) between 1896 and 2005 (tree-ring width in 1/10 mm). (PDF) [file pone.0113507.s001.pdf]

Table S1. Site chronologies of sessile oak (*Quercus petraea*) between 1896 and 2005 (tree-ring width in 1/10 mm). Data represent site chronologies based on 10 trees per sample site; FAL = former arable land (10 sites); FH = former heathland (8 sites); CEF = continuously existing forests (7 sites); NA = not analysed.

Sample site	1	2	3	4	5	6	7	8	9	10	11	12	13	14	15	16	17	18	19	20	21	22	23	24	25
Historical land-use type	FAL	FAL	FAL	FAL	FAL	FAL	FAL	FAL	FAL	FAL	FH	FH	FH	FH	FH	FH	FH	FH	CEF	CEF	CEF	CEF	CEF	CEF	CEF
1896	14	35	24	17	26	NA	22	NA	NA	NA	18	NA	20	20	13	NA	NA	19	17	NA	16	13	17	16	14
1897	14	33	24	16	25	NA	21	NA	NA	NA	18	NA	16	16	11	NA	NA	16	14	NA	13	12	14	13	13
1898	15	31	25	14	28	NA	25	NA	NA	NA	20	NA	22	18	14	NA	NA	20	16	NA	14	14	16	16	15
1899	13	22	21	15	25	14	19	NA	NA	NA	16	NA	13	19	15	NA	NA	13	16	NA	13	13	15	14	12
1900	13	29	29	15	26	23	23	NA	NA	NA	16	NA	15	26	12	NA	NA	16	16	NA	14	13	15	15	13
1901	15	28	28	16	26	20	21	NA	NA	NA	18	NA	15	24	12	NA	NA	15	17	NA	14	13	16	15	13
1902	13	29	28	13	27	25	25	NA	NA	NA	16	NA	18	23	13	NA	19	16	16	NA	14	12	14	13	13
1903	15	27	25	17	30	25	25	NA	NA	NA	19	NA	18	24	14	NA	18	17	17	NA	15	13	16	15	16
1904	16	28	25	17	28	23	23	NA	NA	NA	17	NA	18	28	12	NA	13	17	19	NA	14	14	16	16	16
1905	14	24	21	14	24	23	22	NA	NA	NA	14	NA	19	23	11	NA	13	15	19	NA	14	13	15	15	14
1906	9	21	19	11	18	20	20	NA	NA	NA	15	NA	20	21	11	NA	15	14	16	NA	12	13	14	16	14
1907	9	21	17	10	16	18	17	NA	NA	NA	12	NA	19	19	11	NA	14	17	15	NA	13	12	13	14	13
1908	13	23	18	12	20	22	19	NA	NA	NA	14	NA	19	14	8	NA	16	16	17	NA	14	12	14	15	14
1909	13	22	18	13	21	25	20	NA	NA	NA	9	7	18	11	9	NA	14	10	15	23	13	11	13	12	13
1910	13	22	20	13	19	20	21	NA	NA	NA	18	5	18	13	15	NA	12	16	15	21	13	11	14	15	13
1911	14	21	17	13	19	20	19	26	28	NA	23	4	16	15	13	NA	13	14	13	18	14	11	14	16	14
1912	13	18	17	10	15	18	18	34	34	NA	20	6	16	15	12	NA	13	15	13	18	16	12	13	15	14
1913	13	18	19	12	16	15	20	24	21	27	19	9	16	17	12	NA	12	15	14	18	14	12	12	15	12
1914	15	24	19	14	19	17	25	33	28	21	16	15	21	19	18	NA	18	15	16	19	15	14	14	17	13
1915	12	18	16	12	16	15	18	27	20	11	15	8	13	15	17	NA	14	11	13	14	12	12	12	20	12
1916	11	11	11	9	13	13	17	26	24	13	9	10	14	9	9	NA	18	11	14	14	14	12	12	19	12
1917	11	17	13	10	14	13	14	18	16	12	13	9	15	10	12	NA	12	10	11	13	10	9	9	15	9
1918	11	22	16	11	19	19	19	26	24	17	17	12	19	15	16	NA	19	17	15	16	16	12	13	19	13
1919	9	15	14	8	14	17	17	27	21	18	16	16	16	14	14	35	19	12	12	15	13	10	11	16	11
1920	10	16	14	10	12	16	18	20	18	15	14	12	16	16	17	20	18	12	13	16	13	10	13	15	12
1921	9	14	12	9	9	16	14	18	15	11	13	8	13	13	15	24	12	9	11	14	9	9	10	13	11
1922	10	16	12	10	10	19	15	24	20	14	16	10	15	17	16	30	20	12	12	18	12	10	12	13	12
1923	9	11	10	9	8	16	14	22	16	13	15	11	18	18	15	24	17	12	12	16	12	10	11	14	12
1924	13	12	8	11	6	17	16	34	25	19	20	12	24	22	18	36	20	16	17	21	17	15	14	18	14
1925	8	7	6	6	5	14	11	30	21	15	18	11	13	18	16	27	16	14	14	17	14	14	12	15	13
1926	7	10	9	5	9	13	8	24	24	23	14	12	10	15	14	32	15	13	13	15	13	12	10	16	12
1927	9	11	13	5	8	15	9	23	27	39	12	15	9	19	14	42	14	17	13	17	18	14	13	16	12
1928	8	10	9	5	6	10	7	21	24	23	11	8	8	14	9	19	13	14	11	13	14	11	11	13	9
1929	11	16	13	9	7	18	12	25	25	25	13	9	13	12	11	25	13	16	10	14	14	11	12	12	10
1930	9	15	16	7	10	16	12	21	21	21	13	9	11	11	11	18	11	12	9	14	12	11	12	14	9
1931	13	24	27	11	23	31	23	28	33	35	17	16	19	15	14	27	18	18	13	20	18	14	16	19	13
1932	15	32	34	13	35	38	32	26	32	30	21	16	25	18	20	28	20	21	15	23	18	16	18	22	17
1933	12	29	30	11	33	34	32	29	29	29	21	16	22	19	19	30	18	21	14	19	18	14	17	19	16
1934	11	23	23	10	25	29	26	26	20	17	18	10	17	15	14	19	14	14	12	16	12	11	13	14	12
1935	11	25	24	10	27	28	23	27	24	22	19	11	19	15	18	27	17	15	14	15	14	12	15	15	13
1936	10	20	19	9	24	30	21	22	23	22	21	8	16	14	17	24	18	16	12	16	14	11	14	14	12
1937	12	15	16	11	22	32	23	23	26	29	21	12	16	17	19	27	19	21	12	18	16	13	16	16	13
1938	13	20	17	13	24	29	22	22	26	26	21	14	19	20	24	25	22	22	15	18	17	17	19	17	17
1939	12	23	19	11	22	30	22	20	22	21	21	13	18	17	18	25	20	19	14	15	16	15	15	14	15
1940	9	16	14	8	18	26	19	19	19	17	18	11	16	12	13	18	18	13	12	13	12	10	12	11	12
1941	10	15	13	9	19	24	18	19	20	21	16	13	15	13	15	25	17	12	11	12	13	11	13	13	12
1942	9	18	17	7	20	28	22	17	21	20	19	12	23	15	19	24	17	15	11	12	13	11	12	13	13
1943	6	13	15	7	15	27	19	14	12	20	14	17	16	9	19	22	15	14	8	10	10	9	10	12	10
1944	7	13	14	6	14	26	18	15	12	18	14	18	13	9	14	24	13	12	7	7	9	7	10	7	8
1945	8	14	17	7	14	22	17	15	14	18	15	19	12	11	13	27	13	14	10	11	10	8	11	9	10
1946	8	19	21	8	20	23	21	20	21	16	18	17	17	14	12	25	18	16	13	15	13	11	13	11	14
1947	7	22	24	7	22	23	18	20	20	13	16	15	17	12	12	26	13	13	10	14	11	9	11	10	12
1948	7	22	25	7	21	22	18	23	20	14	14	14	17	15	14	23	19	13	13	14	12	10	11	10	13
1949	11	25	25	10	23	21	17	26	24	15	15	16	19	18	16	26	28	18	15	18	16	14	14	13	17
1950	13	29	27	11	25	24	19	27	26	16	18	20	21	20	17	28	29	18	17	22	15	15	15	14	19
1951	12	23	25	10	20	21	16	26	22	17	16	18	19	20	15	26	30	17	16	19	15	14	15	14	16
1952	10	20	22	9	17	20	16	26	19	12	14	12	16	17	13	21	25	13	14	16	13	12	12	12	15
1953	11	22	22	9	19	21	16	25	22	17	14	14	17	17	12	25	24	12	14	15	14	12	13	13	14
1954	10	14	13	10	16	19	14	23	20	17	12	13	13	15	11	20	20	10	11	14	11	10	11	11	12
1955	11	12	15	10	15	21	15	20	22	20	14	17	20	19	16	26	19	15	13	16	14	12	14	13	16
1956	9	10	13	7	8	14	12	10	12	13	11	13	14	17	13	19	10	12	12	13	11	11	10	10	11
1957	8	14	19	6	10	14	11	12	9	15	14	17	15	17	17	23	11	14	15	13	14	14	14	14	14
1958	10	19	26	8	13	16	14	20	12	15	14	21	17	17	14	25	12	14	16	14	14	15	13	13	15
1959	8	12	14	9	12	17	15	14	9	13	11	17	13	14	13	24	10	11	13	10	13	13	11	11	13
1960	6	13	16	7	10	14	12	15	8	10	11	16	11	11	11	18	8	13	14	8	14	13	9	10	11
1961	8	18	26	8	11	13	11	15	10	12	11	15	12	12	12	21	9	12	13	9	12	12	11	10	12
1962	12	22	31	15	17	17	14	23	18	16	14	16	17	15	14	27	13	14	14	14	15	14	14	12	14
1963	12	24	31	16	19	19	17	26	21	17	12	13	18	13	12	26	15	12	14	15	13	14	13	10	13
1964	10	25	31	16	21	20	17	25	20	16	12	11	21	14	13	25	14	12	17	15	17	15	16	13	16
1965	11	18	25	14	19	18	15	21	20	16	13	12	21	14	14	25	15	15	16	15	19	16	16	14	17
1966	11	15	20	16	18	18	15	24	21	16	13	13	23	16	15	26	16	16	16	18	19	17	14	14	17
1967	11	15	20	17	14	20	16	24	21	15	15	16	24	17	17	21	17	18	20	17	22	18	17	17	21
1968	11	24	34	18	15	18	15	20	19	14	16	21	26	17	16	23	17	18	18	17	19	18	15	16	18
1969	14	23	30	21	15	17	14	22	18	12	15	23	22	16	15	22	17	14	19	15	17	15	16	15	17
1970	12	16	22	17	13	14	11	16	13	10	12	19	19	13	13	18	13	10	17	12	14	11	15	10	12
1971	13	13	19	15	11	15	11	14	12	12	11	21	19	13	15	18	11	12	20	12	15	15	16	12	15
1972	14	13	18	14	10	13	10	14	10	13	13	20	18	12	13	18	12	11	19	11	13	14	15	11	13
1973	20	17	24	16	12	14	11	15	12	14	24	19	16	12	12	17	13	11	18	12	12	17	16	11	13
1974	20	18	20	14	12	11	9	13	11	12	25	18	14	12	13	14	13	13	18	11	13	18	15	11	13
1975	22	15	16	13	10	9	8	13	11	13	24	14	14	10	13	15	12	12	16	10	11	16	14	11	12
1976	19	13	16	11	9	7	7	12	11	13	20	10	13	10	12	12	11	9	15	9	11	14	13	9	11
1977	19	13	14	12	9	6	6	12	12	12	20	8	12	11	12	13	11	10	16	9	13	17	13	10	11
1978	23	17	19	13	13	5	6	14	11	12	24	13	13	12	12	15	12	13	20	10	16	21	16	11	15
1979	25	21	21	14	17	14	7	19	16	21	30	19	19	17	15	21	17	15	24	14	17	21	19	14	16
1980	13	13	15	10	17	14	12	15	17	21	21	21	16	15	16	16	15	15	21	11	17	22	17	16	16
1981	14	13	16	8	20	14	12	13	19	23	28	27	13	12	18	18	20	16	21	10	15	24	11	15	16
1982	16	18	19	7	16	20	14	15	23	25	29	33	19	16	19	19	23	15	16	16	13	21	10	15	15
1983	15	15	17	8	14	17	14	16	16	22	22	27	16	13	17	16	18	11	20	13	13	18	12	14	14
1984	16	12	12	8	13	13	13	14	14	18	20	26	16	13	15	15	12	12	20	13	14	21	12	13	14
1985	21	13	16	12	16	17	13	21	18	19	22	22	20	15	16	18	13	14	21	15	17	22	14	16	16
1986	20	13	15	13	14	17	15	16	16	25	20	24	20	15	20	19	13	13	23	16	18	23	15	16	16
1987	14	11	13	11	13	11	12	14	14	19	19	20	16	13	15	16	10	12	21	13	16	20	11	13	15
1988	19	19	25	14	23	21	18	15	17	21	24	26	25	17	17	21	19	19	25	16	21	24	16	16	19
1989	20	20	23	15	20	25	22	20	22	25	23	25	20	14	17	19	18	16	22	15	17	19	15	16	18
1990	21	24	26	18	21	24	20	21	25	27	24	22	20	17	21	21	15	20	26	14	19	23	15	15	21
1991	23	24	28	18	22	23	18	20	22	27	24	23	21	17	21	24	17	19	20	12	18	19	16	17	20
1992	18	21	23	16	17	23	18	18	20	24	20	20	17	15	17	19	15	14	17	12	14	16	13	13	15
1993	20	19	18	16	18	23	19	22	24	27	20	21	21	14	17	19	18	16	19	12	14	16	14	12	14
1994	15	17	16	12	15	19	16	21	22	24	17	21	19	16	16	15	16	12	14	12	11	15	13	12	15
1995	15	14	16	11	14	17	16	18	21	22	16	18	17	14	14	15	14	13	16	12	13	15	14	12	15
1996	14	10	10	7	13	19	13	11	12	17	14	20	16	13	12	15	13	12	14	11	13	14	12	10	12
1997	15	14	10	9	15	26	19	15	18	21	17	23	21	16	18	20	18	15	20	15	16	18	16	12	16
1998	16	17	14	13	18	25	20	18	20	20	17	24	22	15	18	19	18	18	21	13	15	19	18	15	17
1999	20	20	20	17	20	23	23	22	27	26	19	27	23	17	21	21	20	17	22	19	18	20	19	16	17
2000	16	16	16	13	19	22	18	18	22	20	15	25	18	15	19	18	19	14	19	15	13	15	16	12	15
2001	20	23	22	17	26	30	24	27	30	27	18	30	25	18	18	24	24	16	20	19	15	20	19	15	16
2002	19	21	24	17	25	28	18	20	27	23	19	27	25	16	17	24	19	19	27	23	15	21	17	16	18
2003	17	20	18	15	22	23	18	20	26	21	17	26	20	16	17	24	18	16	23	18	13	19	17	13	15
2004	16	16	16	13	18	19	15	18	25	21	15	23	18	16	18	21	16	14	20	16	12	15	17	13	15
2005	18	19	17	13	19	22	15	20	24	19	18	28	18	15	19	24	17	14	17	19	13	14	15	13	13
