# Supplementary material for: Does Forest Continuity Enhance the Resilience of Trees to Environmental Change?
Source: PLoS One. 2014 Dec 10;9(12):e113507. doi: 10.1371/journal.pone.0113507 (PMC4262476; doi:10.1371/journal.pone.0113507)
Supplement: Table S2 — Mean monthly precipitation and precipitation total of the growing season (April–October, rainvp) (in mm) at the weather station Wilsede (Lüneburg Heath, NW Germany) for the period 1896 to 2005. (PDF) [file pone.0113507.s002.pdf]

Table S2. Mean monthly precipitation and precipitation total of the growing season (April-October, rainvp) (in mm) at the weather station Wilsede (Lüneburg Heath, NW Germany) for the period 1896 to 2005 (Source: German Weather Service (DWD, Hamburg, Germany) and Hamburger Bildungsserver (HBS, Hamburg, Germany)).

Year	Jan	Feb	Mar	Apr	May	Jun	Jul	Aug	Sep	Oct	Nov	Dec	rainvp
1896	61	17	100	77	21	43	105	131	129	43	31	31	465
1897	60	55	85	64	74	38	55	56	90	53	41	50	368
1898	45	80	127	32	128	130	149	57	34	65	7	64	589
1899	99	31	26	69	109	35	95	51	107	32	53	45	439
1900	91	36	21	54	28	139	144	68	35	124	55	62	537
1901	56	53	50	68	18	42	106	72	106	93	167	69	421
1902	85	28	72	43	114	110	177	102	60	78	4	61	659
1903	53	58	42	67	84	75	99	133	96	95	84	9	587
1904	53	66	21	48	68	71	60	48	90	62	109	53	401
1905	73	54	84	73	26	89	168	63	83	166	49	62	577
1906	95	63	87	34	71	51	115	141	87	38	78	80	513
1907	87	62	53	11	51	99	85	76	46	30	28	83	400
1908	38	63	55	125	116	55	76	78	51	4	34	8	405
1909	35	86	33	54	29	47	127	112	163	65	100	76	528
1910	84	90	57	34	60	111	172	169	48	14	72	34	594
1911	28	103	47	15	45	47	39	23	28	74	41	67	255
1912	77	59	56	35	83	139	93	140	62	51	124	78	588
1913	72	35	56	31	47	58	75	49	55	74	81	93	357
1914	37	43	120	32	60	67	149	37	77	72	40	37	462
1915	108	21	154	57	21	19	109	58	30	59	49	121	292
1916	100	53	49	50	42	74	99	83	31	64	75	71	399
1917	87	13	32	63	17	39	85	139	13	108	55	52	397
1918	135	55	8	58	26	70	95	145	73	65	16	108	474
1919	37	28	74	65	20	42	93	46	45	75	142	135	314
1920	87	45	17	39	77	17	119	112	101	32	4	51	462
1921	162	18	12	34	31	98	20	44	38	53	58	61	290
1922	84	31	31	56	88	63	99	77	93	127	94	56	544
1923	76	58	31	31	86	66	80	76	66	140	65	39	513
1924	19	37	28	77	61	49	75	198	145	48	22	13	578
1925	72	43	85	31	24	43	21	84	106	68	66	136	337
1926	108	82	74	20	88	87	277	72	93	169	124	67	778
1927	50	22	32	106	31	124	98	134	77	35	52	27	508
1928	65	50	19	71	94	71	59	103	13	119	96	60	470
1929	41	27	11	69	18	43	45	4	46	108	25	100	251
1930	19	17	32	59	74	61	64	101	111	68	161	20	480
1931	92	28	24	76	34	89	200	144	125	59	28	39	649
1932	79	24	24	81	66	14	55	68	97	120	46	9	409
1933	41	40	36	33	33	186	61	59	72	77	49	34	495
1934	45	49	24	28	36	34	60	23	56	84	89	59	286
1935	76	112	25	98	63	71	90	55	108	87	25	32	470
1936	35	111	12	122	48	29	91	78	177	116	46	45	517
1937	61	102	70	48	44	67	53	43	49	32	50	52	293
1938	131	26	20	20	50	18	89	65	136	127	66	45	466
1939	68	31	56	60	41	40	99	135	25	132	127	34	469
1940	22	27	103	75	23	53	140	90	69	46	92	95	419
1941	51	62	46	33	74	47	98	159	58	189	46	84	617
1942	23	22	17	38	57	48	180	23	79	152	93	27	523
1943	42	66	15	107	30	102	53	128	55	10	65	29	390
1944	88	37	64	39	58	84	96	20	32	72	156	42	369
1945	30	61	27	69	58	51	105	166	28	49	58	84	471
1946	38	179	34	15	98	111	51	97	107	66	37	18	543
1947	20	12	52	57	38	70	54	42	44	26	170	132	280
1948	118	73	18	35	78	46	160	111	45	25	37	11	479
1949	51	32	59	73	79	67	51	43	56	7	106	81	319
1950	43	75	17	68	70	67	118	56	76	80	74	31	469
1951	84	48	57	38	74	66	65	70	79	1	117	38	369
1952	83	23	82	31	50	67	31	86	52	91	120	47	378
1953	30	49	18	35	42	92	66	38	89	36	32	36	367
1954	68	14	31	33	27	31	219	140	65	107	50	98	579
1955	22	50	30	51	57	114	120	50	75	71	74	64	491
1956	47	28	62	39	16	139	180	84	30	94	53	51	544
1957	45	58	83	33	36	74	132	51	183	32	56	93	497
1958	70	101	18	60	70	105	162	122	30	91	27	55	592
1959	64	6	21	42	9	16	28	70	0	33	61	28	158

Year	Jan	Feb	Mar	Apr	May	Jun	Jul	Aug	Sep	Oct	Nov	Dec	rainvp
1960	77	36	24	43	86	29	58	160	39	145	96	118	518
1961	80	67	54	68	103	48	129	137	25	62	80	80	501
1962	84	62	36	59	88	41	132	76	47	14	13	53	398
1963	18	22	45	46	40	72	36	143	100	44	126	17	436
1964	27	30	37	37	109	47	51	168	87	63	52	56	523
1965	66	34	33	91	105	63	163	55	37	15	62	121	438
1966	71	80	60	56	71	122	114	102	53	63	63	121	526
1967	85	57	92	41	68	59	108	90	75	75	52	105	474
1968	78	30	61	28	79	115	94	69	140	91	48	22	588
1969	70	67	22	76	77	119	29	52	8	41	62	29	325
1970	24	99	79	93	66	84	96	57	90	98	98	34	490
1971	28	34	21	7	28	169	61	61	51	29	100	86	399
1972	7	14	43	60	90	113	46	58	63	33	137	15	402
1973	36	34	25	72	52	54	95	28	62	100	134	86	391
1974	84	33	29	28	55	54	117	61	75	101	71	195	463
1975	57	13	58	89	56	24	41	42	57	50	68	34	270
1976	137	34	33	21	53	46	33	35	69	36	73	38	271
1977	71	70	55	131	31	42	93	68	46	63	99	44	343
1978	77	22	91	11	28	206	72	69	164	44	30	114	582
1979	51	45	76	80	80	87	114	92	40	20	91	106	432
1980	40	87	30	67	19	154	162	89	98	66	75	72	587
1981	94	44	146	17	58	98	59	84	168	127	97	69	595
1982	86	6	78	54	88	88	25	60	22	102	55	74	385
1983	127	73	95	99	71	30	24	10	54	91	80	68	281
1984	114	58	24	18	111	82	103	29	128	94	56	47	547
1985	55	19	51	79	28	120	54	94	79	16	80	89	392
1986	118	10	61	27	53	63	54	80	60	59	65	137	369
1987	97	38	46	39	71	85	83	84	114	60	108	56	498
1988	100	88	123	8	25	62	124	52	55	53	64	81	370
1989	17	53	62	68	18	53	59	157	25	84	37	89	395
1990	53	78	56	35	41	87	23	77	201	53	111	79	482
1991	49	27	15	41	73	162	49	82	48	35	87	85	449
1992	48	53	96	95	41	21	57	97	45	96	117	60	357
1993	111	32	21	47	91	72	171	80	93	59	29	175	566
1994	136	24	130	85	61	102	46	82	72	70	86	106	433
1995	124	108	83	54	59	78	22	38	120	22	72	25	339
1996	2	64	13	24	60	29	59	42	43	98	93	40	331
1997	13	85	47	41	121	81	74	49	35	58	49	73	418
1998	112	25	84	121	44	0	114	76	70	164	66	96	468
1999	78	81	58	51	44	54	78	75	74	52	31	76	376
2000	65	85	137	23	46	57	129	60	72	42	37	68	406
2001	55	71	53	70	44	106	80	76	237	35	96	117	578
2002	87	147	59	69	76	79	91	68	18	32	74	9	364
2003	59	7	37	51	75	42	38	44	51	47	53	64	296
2004	94	86	50	34	32	110	94	62	93	37	90	18	427
2005	94	57	60	32	93	49	153	118	64	43	70	53	519
