# Supplementary material for: Does Forest Continuity Enhance the Resilience of Trees to Environmental Change?
Source: PLoS One. 2014 Dec 10;9(12):e113507. doi: 10.1371/journal.pone.0113507 (PMC4262476; doi:10.1371/journal.pone.0113507)
Supplement: Table S3 — Mean monthly temperature and mean annual temperature (mean temp) (in °C) at the weather station Wilsede (Lüneburg Heath, NW Germany) for the period 1896 to 2005. (PDF) [file pone.0113507.s003.pdf]

Table S3. Mean monthly temperature and mean annual temperature (mean temp) (in °C) at the weather station Wilsede (Lüneburg Heath, NW Germany) for the period 1896 to 2005 (Source: German Weather Service (DWD, Hamburg, Germany) and Hamburger Bildungsserver (HBS, Hamburg, Germany)).

Year	Jan	Feb	Mar	Apr	Mai	Jun	Jul	Aug	Sep	Oct	Nov	Dec	mean temp
1896	1,1	1,6	6,4	7,1	11,4	17,8	17,8	15,0	14,0	10,0	1,7	1,1	8,8
1897	-2,9	1,6	6,0	7,8	11,5	17,7	16,3	18,1	13,2	8,4	4,0	3,4	8,8
1898	4,7	2,9	3,7	7,9	11,8	15,4	14,2	18,9	14,7	9,2	5,8	5,5	9,6
1899	3,6	3,4	3,5	8,0	11,5	15,5	18,1	17,4	13,0	8,6	8,4	-1,9	9,1
1900	2,1	1,7	2,2	7,4	12,0	16,5	18,9	16,9	14,3	9,9	6,1	4,5	9,4
1901	-2,7	-2,0	3,4	9,0	13,7	16,1	19,4	16,9	14,1	10,5	4,1	2,3	8,7
1902	4,3	-1,2	4,2	8,0	9,3	16,6	15,5	14,8	12,3	7,7	2,8	-1,0	7,8
1903	2,5	5,5	7,4	5,3	13,5	15,2	16,7	15,5	14,5	10,7	5,5	0,4	9,4
1904	0,1	2,2	3,8	9,5	13,0	15,2	18,8	17,0	12,6	8,9	4,7	4,0	9,2
1905	0,1	2,7	5,5	6,0	13,0	18,1	18,5	17,0	13,2	5,5	3,9	2,3	8,8
1906	2,6	2,1	3,3	9,2	14,2	15,7	17,7	16,6	13,0	10,9	7,8	-1,0	9,3
1907	1,1	-0,3	4,0	7,2	13,6	14,8	15,0	16,1	14,0	12,8	4,5	2,8	8,8
1908	-0,2	3,1	3,6	6,2	13,6	17,8	17,7	15,4	13,3	9,4	2,8	1,1	8,7
1909	-0,3	-1,0	2,4	8,4	11,0	14,6	15,4	16,6	13,1	11,8	3,3	3,1	8,2
1910	3,1	4,4	4,8	8,1	13,4	17,3	15,9	17,2	13,5	10,2	3,4	4,1	9,6
1911	1,3	3,2	5,3	9,1	14,9	16,3	20,3	20,9	15,3	9,4	5,9	4,4	10,5
1912	-1,7	3,8	7,2	8,1	12,1	16,2	19,5	14,7	10,3	7,6	4,5	5,4	9,0
1913	1,0	3,4	7,2	9,4	13,6	15,8	15,6	15,6	13,6	10,8	8,1	3,3	9,8
1914	-1,0	5,6	5,8	10,8	11,6	15,2	19,0	18,4	13,6	9,6	4,8	5,3	9,9
1915	1,5	2,4	2,9	7,2	13,5	18,2	16,4	16,1	13,4	7,0	2,4	4,7	8,8
1916	5,0	1,9	3,8	9,4	13,8	13,1	16,1	16,7	13,0	9,6	5,9	2,8	9,3
1917	-1,5	-2,2	0,7	5,1	16,6	20,9	18,5	18,0	16,0	8,4	6,9	-1,6	8,8
1918	2,5	3,4	5,3	9,6	14,9	13,6	17,1	16,3	13,3	9,6	4,3	5,6	9,6
1919	2,0	0,8	4,1	7,0	13,6	15,8	15,2	16,7	15,9	7,0	0,6	1,7	8,4
1920	3,5	5,0	7,9	10,1	13,9	16,2	18,7	15,4	13,4	7,8	2,7	1,5	9,7
1921	5,4	2,4	7,1	8,6	14,9	14,7	18,7	17,7	13,9	12,1	0,5	2,2	9,9
1922	-1,8	-0,3	4,3	5,9	14,2	15,7	16,0	15,5	12,0	5,1	4,1	4,3	7,9
1923	3,4	1,0	5,7	7,3	11,5	11,4	18,9	15,5	13,8	11,3	3,8	-0,9	8,6
1924	-1,3	-1,5	2,9	6,8	14,5	15,8	17,3	15,0	14,2	10,3	4,2	3,6	8,5
1925	4,2	4,9	2,5	9,1	14,9	15,6	19,1	17,7	11,6	9,2	2,9	0,7	9,4
1926	0,9	4,9	4,9	10,6	10,8	14,6	18,2	16,6	15,4	8,2	6,9	2,2	9,5
1927	3,4	2,3	7,2	7,4	10,8	13,8	17,8	17,2	14,2	9,9	2,8	-2,1	8,7
1928	2,8	3,6	3,5	8,0	10,7	14,0	18,1	16,2	12,8	9,6	7,5	1,0	9,0
1929	-3,6	-9,2	3,3	4,9	13,8	14,9	17,5	18,1	16,4	10,3	6,1	4,8	8,1
1930	4,3	0,9	4,6	8,8	12,2	18,7	17,0	16,7	13,6	9,9	6,8	1,7	9,6
1931	3,1	0,6	2,2	6,2	14,9	16,5	16,5	15,1	10,5	8,1	4,7	1,5	8,3
1932	4,8	0,3	2,4	6,8	12,1	15,0	17,2	18,3	14,8	9,1	4,7	1,0	8,9
1933	-1,2	1,8	5,0	7,4	11,8	14,5	18,3	17,3	13,9	9,4	3,1	-3,6	8,1
1934	2,6	1,8	4,0	9,8	13,4	16,6	18,3	16,1	15,3	10,2	3,9	11,0	10,3
1935	1,8	2,7	3,9	7,2	11,0	16,7	18,2	16,6	13,6	9,3	5,5	2,9	9,1
1936	7,7	1,7	5,1	6,3	13,0	15,9	16,4	16,3	13,1	7,7	4,6	4,0	9,3
1937	2,6	3,2	3,6	7,9	14,8	16,5	17,5	17,5	13,4	9,8	4,1	2,1	9,4
1938	5,1	1,9	6,4	5,7	10,9	16,6	16,6	17,2	13,7	9,3	6,8	-1,1	9,1
1939	7,4	1,8	2,9	8,4	10,6	16,5	16,9	17,1	13,4	7,8	6,2	0,3	9,1
1940	-12,3	-1,0	3,8	8,0	12,6	16,7	16,0	14,8	13,0	8,4	6,1	-2,3	7,0
1941	-4,6	1,5	4,5	6,4	9,5	16,6	18,4	14,8	12,6	9,1	3,5	4,5	8,1
1942	-9,1	-2,7	3,0	8,3	12,2	14,9	16,1	17,2	15,2	12,2	4,3	3,7	7,9
1943	1,4	2,9	5,4	8,9	13,0	14,2	17,9	17,7	13,7	10,6	3,4	2,1	9,3
1944	6,8	0,6	2,5	8,9	12,4	14,1	16,9	19,7	12,4	10,0	4,6	1,3	9,2
1945	-6,0	3,7	5,5	8,9	14,0	16,9	18,4	16,1	14,7	10,6	4,5	4,0	9,3
1946	-1,7	3,4	4,3	9,6	13,7	14,6	17,9	16,3	14,0	8,5	4,4	-1,9	8,6
1947	-4,2	-3,2	3,3	9,3	14,5	18,5	19,2	19,6	16,7	8,4	5,6	4,9	9,4
1948	8,0	1,9	5,7	9,3	13,7	16,0	16,1	15,9	13,2	9,5	3,9	1,0	9,5
1949	3,7	1,6	3,5	10,0	12,1	14,5	18,7	16,8	16,0	11,1	4,0	6,6	9,9
1950	0,9	3,3	4,8	6,8	13,7	17,6	18,3	17,0	13,0	8,5	4,7	-1,3	9,0
1951	2,4	2,2	2,7	7,5	11,8	15,5	16,9	17,3	14,6	7,3	8,2	4,2	9,2
1952	1,5	1,4	2,5	11,1	12,5	14,9	17,7	17,4	11,1	7,5	1,8	0,4	8,3
1953	1,0	0,7	4,6	9,1	13,5	16,8	17,4	16,9	13,2	10,9	6,5	4,1	9,6
1954	-2,3	-3,6	4,2	6,4	13,1	16,2	14,4	16,4	13,8	11,0	5,2	4,4	8,3
1955	-1,2	-1,8	0,3	7,5	10,1	14,6	18,0	17,5	13,8	8,8	4,9	3,8	8,0
1956	0,9	-9,3	3,3	5,0	13,1	13,4	17,0	14,1	13,9	8,9	3,8	3,6	7,3
1957	2,2	4,6	6,6	7,7	10,3	17,1	17,6	15,5	11,7	9,9	5,2	1,0	9,1
1958	0,6	2,4	0,3	5,5	13,1	14,6	16,9	17,1	14,7	10,6	4,4	3,6	8,7
1959	0,5	0,2	6,3	9,8	13,4	16,7	20,1	18,3	13,9	9,4	4,2	2,8	9,6

Year	Jan	Feb	Mar	Apr	Mai	Jun	Jul	Aug	Sep	Oct	Nov	Dec	mean temp
1960	1,2	1,0	4,3	7,7	12,9	16,4	15,6	15,7	13,1	9,7	7,2	2,0	8,9
1961	0,2	5,6	6,4	10,6	10,4	16,4	15,3	15,9	16,7	11,0	4,3	-0,3	9,4
1962	3,0	1,6	0,6	8,4	10,0	14,1	14,6	15,1	12,3	9,5	3,5	-2,6	7,5
1963	-7,5	-6,1	3,0	8,9	12,5	16,7	17,5	16,3	13,6	8,5	8,0	-2,4	7,4
1964	-1,6	0,9	0,8	9,0	13,9	16,9	17,9	16,3	13,9	7,4	5,2	1,7	8,5
1965	2,4	0,1	3,1	7,3	11,7	15,6	15,0	15,0	13,5	8,8	0,8	3,3	8,1
1966	-1,6	2,1	4,3	8,6	13,4	17,5	16,1	15,9	13,3	11,3	3,0	3,0	8,9
1967	2,6	4,0	6,2	6,7	12,9	15,0	18,7	16,7	14,3	11,7	4,7	1,9	9,6
1968	-0,3	0,8	5,2	9,7	11,0	16,6	16,3	17,2	14,2	10,9	4,5	-1,5	8,7
1969	2,4	-1,5	-0,3	7,3	13,1	15,6	18,7	17,1	13,9	11,1	5,9	-4,2	8,3
1970	-2,8	-0,7	2,0	5,7	12,3	17,4	16,4	16,8	13,3	9,5	7,1	1,4	8,2
1971	0,5	2,6	2,0	7,9	14,6	14,5	18,0	17,9	12,6	9,7	4,4	4,8	9,1
1972	-2,2	2,7	5,7	7,9	12,1	14,7	18,0	16,0	10,9	7,4	5,5	2,3	8,4
1973	1,7	2,2	4,9	5,4	12,4	16,7	17,7	17,8	14,3	8,0	4,0	1,6	8,9
1974	4,8	4,2	5,6	8,1	11,4	14,8	15,6	17,0	13,9	6,4	6,5	6,4	9,6
1975	6,4	2,1	4,3	7,3	11,6	15,4	18,9	19,9	15,8	8,1	4,2	2,9	9,7
1976	1,9	1,3	1,4	7,1	13,0	17,3	19,3	16,2	13,1	9,7	5,9	0,1	8,9
1977	1,5	4,1	6,4	6,1	12,0	15,6	16,8	16,2	12,3	11,2	6,2	3,6	9,3
1978	1,9	-0,9	6,0	6,6	12,5	15,4	15,8	15,6	12,6	10,3	5,7	0,1	8,5
1979	-3,8	-3,1	3,8	7,3	12,9	16,3	15,4	16,1	13,4	8,8	4,8	4,8	8,1
1980	-1,8	2,5	3,5	7,0	11,0	15,2	15,6	16,8	14,7	8,7	4,1	2,4	8,3
1981	0,2	0,6	7,5	7,9	14,4	15,4	16,7	16,7	14,3	8,4	5,5	-2,4	8,8
1982	-0,7	1,3	4,8	7,3	12,9	16,5	19,3	18,0	16,2	10,7	7,3	3,1	9,7
1983	5,4	-0,7	5,4	9,2	12,0	16,6	20,3	18,8	14,5	10,0	4,5	2,3	9,9
1984	2,3	0,9	2,6	7,9	11,9	14,2	16,0	17,9	13,1	11,2	5,8	2,4	8,9
1985	-5,0	-2,0	3,4	8,4	14,3	14,2	17,5	16,5	13,6	9,7	1,7	5,3	8,1
1986	0,8	-6,8	3,8	6,7	14,4	16,4	17,7	16,5	11,4	10,5	7,7	3,3	8,5
1987	-6,4	0,3	-0,5	10,0	10,3	14,4	17,3	15,8	14,3	9,7	6,3	3,5	7,9
1988	5,1	3,5	3,5	8,1	15,2	15,2	17,7	17,3	13,7	9,7	4,4	4,6	9,8
1989	4,1	4,8	7,8	7,5	14,1	16,3	18,1	17,6	15,1	11,5	3,3	3,5	10,3
1990	4,8	7,1	7,6	8,1	13,6	15,8	16,8	18,8	12,1	10,8	5,4	2,5	10,3
1991	3,0	-2,1	7,7	7,9	10,4	13,5	19,5	17,3	14,9	8,8	4,7	2,3	9,0
1992	2,0	4,4	5,6	8,6	15,0	18,5	19,4	19,0	13,9	6,9	6,4	2,4	10,2
1993	3,1	0,0	4,3	10,9	14,8	15,6	16,2	15,5	12,3	8,7	0,7	4,1	8,9
1994	4,3	-0,7	6,6	9,2	12,9	15,6	21,8	17,9	13,4	8,0	7,9	4,8	10,1
1995	1,4	5,7	3,8	8,7	12,6	14,8	20,5	19,3	13,7	12,6	4,4	-2,0	9,6
1996	-3,2	-2,2	1,2	9,2	11,3	15,2	16,3	18,2	11,5	9,9	5,3	-1,6	7,6
1997	-1,6	5,6	6,3	7,0	12,8	16,6	17,8	20,6	14,0	8,3	4,8	3,0	9,6
1998	4,2	6,4	6,1	9,5	14,4	16,5	16,3	16,8	14,1	9,1	2,5	2,4	9,9
1999	4,3	2,0	6,4	9,9	13,9	15,8	19,8	17,4	18,0	10,0	5,2	4,0	10,6
2000	3,1	5,2	5,8	10,7	15,4	17,0	15,7	17,4	14,8	11,5	7,9	4,4	10,7
2001	1,8	2,9	3,7	7,9	14,1	14,2	19,0	19,2	12,6	13,5	5,6	0,9	9,6
2002	3,3	6,1	5,9	8,4	14,4	17,1	17,8	19,7	14,2	8,5	5,7	-0,4	10,1
2003	1,3	-1,1	5,6	9,0	14,1	18,7	19,5	20,2	14,5	6,2	7,7	3,1	9,9
2004	1,6	3,8	5,2	10,0	12,0	15,6	16,7	19,3	14,8	10,8	5,3	3,0	9,8
2005	3,9	0,5	4,6	10,1	13,0	15,9	18,5	16,0	15,7	11,9	5,8	2,6	9,9
