# Supplementary material for: Does Forest Continuity Enhance the Resilience of Trees to Environmental Change?
Source: PLoS One. 2014 Dec 10;9(12):e113507. doi: 10.1371/journal.pone.0113507 (PMC4262476; doi:10.1371/journal.pone.0113507)
Supplement: Table S4 — Mean tree-ring width (TRW) of sessile oak ( Quercus petraea ) during the period 1896–2005 (in 1/10 mm) and soil parameters. (PDF) [file pone.0113507.s004.pdf]

Table S4. Mean tree-ring width (TRW) of sessile oak (*Quercus petraea*) during the period 1896-2005 (in 1/10 mm) and soil parameters. Mean TRW per sample site is based on the data given in Table S1. Soil data are available for 21 sample sites (data from von Oheimb et al. [15]). Abbreviations: FAL = former arable land; FH = former heathland; CEF = continuously existing forests; P_{pa} = plant available phosphorous; P_t = total P-content; CEC = cation exchange capacity; O = organic layer, A = A-horizon (i.e. upper mineral horizon); NA = not analysed.

Sample site		1	2	3	4	5	6	7	8	9	10	11	12	13	14	15	16	17	18	19	20	21	22	23	24	25
Historical land-use type		FAL	FAL	FAL	FAL	FAL	FAL	FAL	FAL	FAL	FAL	FH	FH	FH	FH	FH	FH	FH	FH	CEF	CEF	CEF	CEF	CEF	CEF	CEF
Mean TRW		13.16	18.68	19.22	11.64	17.39	19.64	16.84	20.44	19.78	18.87	17.06	16.43	17.45	15.58	14.85	22.25	16.08	14.54	15.84	14.79	14.26	14.36	13.88	13.84	13.96
C-content (%)	O	11.47	2.90	4.09	NA	11.56	11.38	NA	3.76	13.18	11.77	10.00	19.55	26.05	10.51	NA	NA	26.81	25.47	26.63	13.38	26.06	28.42	13.91	9.42	15.92
	A	2.89	1.78	1.86	NA	2.55	2.05	NA	1.49	1.49	0.92	1.11	1.18	7.84	2.13	NA	NA	1.22	1.00	2.28	4.77	2.63	2.06	1.08	0.95	1.33
N-content (%)	O	0.60	0.17	0.23	NA	0.61	0.59	NA	0.22	0.62	0.51	0.49	0.87	1.36	0.54	NA	NA	1.21	0.98	0.98	0.61	1.15	1.14	0.61	0.45	0.67
	A	0.15	0.11	0.11	NA	0.13	0.10	NA	0.09	0.07	0.03	0.05	0.04	0.37	0.09	NA	NA	0.05	0.04	0.07	0.19	0.09	0.06	0.04	0.04	0.04
C/N-ratio	O	19.4	17.7	17.7	NA	19.1	19.4	NA	17.3	20.6	17.9	20.4	22.8	19.1	19.5	NA	NA	22.2	26.1	27.4	22.1	22.6	25.5	23.4	20.9	24.6
	A	20.5	16.4	17.0	NA	19.8	20.8	NA	17.7	22.8	28.6	25.0	29.1	20.9	25.1	NA	NA	26.8	31.1	35.7	25.9	29.0	35.9	28.9	23.8	28.4
P_{pa} (mg L^{-1})	O	7.1	2.9	2.6	NA	5.3	5.9	NA	5.8	6.4	9.6	7.1	5.5	8.7	4.1	NA	NA	7.5	6.4	4.6	9.4	9.0	9.2	6.7	5.2	5.0
	A	8.3	20.2	30.8	NA	27.3	20.1	NA	27.3	10.1	14.0	4.9	3.7	14.7	4.7	NA	NA	3.5	3.4	2.5	29.1	6.2	5.8	2.4	3.5	1.5
P_t (mg L^{-1})	O	77.8	40.6	44.9	NA	72.9	79.0	NA	38.1	73.7	48.2	67.1	80.9	188.6	65.1	NA	NA	153.9	122.8	105.2	70.8	175.5	175.2	93.0	50.7	63.4
	A	171.5	340.1	376.7	NA	294.5	301.9	NA	228.5	202.9	125.0	73.6	57.0	487.0	131.6	NA	NA	57.3	86.6	94.9	198.1	159.5	87.2	80.6	70.5	67.1
C/P-ratio	O	221.3	107.0	136.6	NA	237.8	216.1	NA	148.1	268.1	366.7	223.7	362.4	207.2	241.9	NA	NA	261.3	311.1	379.7	283.5	222.7	243.3	224.4	278.8	376.8
	A	218.9	68.1	64.1	NA	112.7	88.3	NA	84.9	95.3	95.5	196.5	269.3	209.2	210.7	NA	NA	275.8	150.5	312.2	313.2	214.5	307.3	173.4	174.8	257.9
Base saturation (%)	O	42.8	61.5	29.0	NA	34.7	41.1	NA	52.5	45.4	20.4	47.4	26.9	28.7	60.4	NA	NA	31.4	28.7	23.0	29.7	30.4	17.3	21.5	21.9	20.9
	A	37.2	61.4	0.0	NA	38.8	51.7	NA	51.0	52.6	41.7	0.0	30.0	25.2	58.2	NA	NA	50.0	62.5	32.4	23.0	13.6	13.8	30.3	0.0	52.9
CEC (mval L^{-1})	O	21.8	9.8	9.3	NA	22.5	28.8	NA	15.2	30.8	28.7	23.4	35.1	41.3	25.4	NA	NA	23.0	37.7	56.7	31.8	51.8	53.7	40.5	17.1	33.8
	A	12.9	6.6	3.3	NA	7.4	8.7	NA	7.7	5.7	7.2	2.0	1.5	20.3	8.3	NA	NA	4.2	7.2	11.1	15.0	12.2	9.8	5.0	0.5	2.6
pH_{H2O}	O	4.0	4.5	4.1	NA	3.7	4.5	NA	4.4	4.6	4.0	5.0	4.3	3.7	5.0	NA	NA	3.9	3.5	4.0	3.7	4.1	3.8	4.2	4.2	3.9
	A	3.9	4.5	4.0	NA	3.7	4.1	NA	4.1	4.2	3.9	4.2	4.2	3.6	4.9	NA	NA	3.9	3.9	4.0	3.6	3.8	3.9	4.1	4.0	3.9
